# Supplementary material for: Patients’ Engagement in Early Detection of COVID-19 Symptoms: An Observational Study in the Very Early Peak of the Pandemic in Italy in 2020
Source: Int J Environ Res Public Health. 2022 Mar 5;19(5):3058. doi: 10.3390/ijerph19053058 (PMC8910395; doi:10.3390/ijerph19053058)
Supplement: Supplementary file 1 [file ijerph-19-03058-s001.zip › ijerph-1606413-supplementary.pdf]

Supplementaty Table S1: results from logistic regressions (univariate and multivariate) for the three groups										
	**Blood donors (n=659)**				**Professionals (n=209)**				**Volunteers (n=91)**	
	**Univariate analysis**		**Multivariate analysis[1]**		**Univariate analysis**		**Multivariate analysis[1]**		**Univariate analysis**	
**Variable**	**OR (95% CI)**	**p-Value**	**OR (95% CI)**	**p-Value**	**OR (95% CI)**	**p-Value**	**OR (95% CI)**	**p-Value**	**Odds Ratio (95% CI)**	**p-Value**
**Age**										
<55	1.00 (Reference)				1.00 (Reference)				1.00 (Reference)	
>55	1.08 (0.69-1.67)	0.747			1.02 (0.51-2.02)	0.957			0.40 (0.13-1.19)	0.100
**Gender**										
Female	1.00 (Reference)				1.00 (Reference)				1.00 (Reference)	
Male	1.14 (0.82-1.58)	0.434			0.70 (0.39-1.26)	0.236			0.57 (0.24-1.32)	0.211
**Lives alone**										
No	1.00 (Reference)				1.00 (Reference)				1.00 (Reference)	
Yes	0.79 (0.50-1.27)	0.334			1.81 (0.86-3.81)	0.116			0.94 (0.33-2.68)	0.911
**Smoking**										
Smoker	1.00 (Reference)		1.00 (Reference)		1.00 (Reference)				1.00 (Reference)	
Non-smoker	2.40 (1.43-4.02)	<0.001	2.61 (1.42-4.80)	0.002	0.75 (0.31-1.80)	0.519			0.35 (0.12-1.04)	0.058
Former smoker	2.04 (1.10-3.76)	0.022	2.09 (1.02-4.27)	0.044	0.58 (0.20-1.64)	0.306			0.44 (0.11-1.87)	0.268
**Comorbidity/chronic condition**										
No	1.00 (Reference)				1.00 (Reference)				1.00 (Reference)	
Yes	0.94 (0.64-1.40)	0.776			0.89 (0.48-1.67)	0.724			0.65 (0.24-1.78)	0.401
**Previous vaccinations**										
No	1.00 (Reference)				1.00 (Reference)				1.00 (Reference)	
Yes	1.11 (0.75-1.63)	0.605			1.50 (0.84-2.68)	0.171			0.81 (0.34-1.95)	0.638
**Diarrhea**										
No	1.00 (Reference)				1.00 (Reference)				1.00 (Reference)	
Yes	1.75 (1.24-2.46)	0.001			1.79 (0.97-3.30)	0.062			4.15 (1.65-10.4)	0.002
**Nausea/vomit**										
No	1.00 (Reference)				1.00 (Reference)				1.00 (Reference)	
Yes	2.85 (1.85-4.40)	<0.001			2.39 (1.15-4.97)	0.019			5.30 (1.65-17.0)	0.005
**Poor appetite**										
No	1.00 (Reference)		1.00 (Reference)		1.00 (Reference)		1.00 (Reference)		1.00 (Reference)	
Yes	6.16 (4.16-9.11)	<0.001	2.32 (1.41-3.84)	<0.001	5.85 (3.04-11.3)	<0.001	3.67 (1.60-8.38)	0.002	5.78 (2.16-15.5)	<0.001
**Smell dysfunctions**										
No	1.00 (Reference)				1.00 (Reference)				1.00 (Reference)	
Yes	6.04 (4.19-8.71)	<0.001			6.94 (3.55 -13.1)	<0.001			6.51 (2.47-17.2)	<0.001
**Taste dysfunction**										
No	1.00 (Reference)		1.00 (Reference)		1.00 (Reference)		1.00 (Reference)		1.00 (Reference)	
Yes	6.27 (4.35-9.04)	<0.001	2.68 (1.15-6.22)	0.021	9.98 (4.97-20.0)	<0.001	8.90 (2.01-37.9)	0.003	9.62 (3.46-26.7)	<0.001
**Dizziness**										
No	1.00 (Reference)		1.00 (Reference)		1.00 (Reference)				1.00 (Reference)	
Yes	2.54 (1.67-3.88)	<0.001	2.10 (1.26-3.50)	0.0046	0.71 (0.34-1.48)	0.363			1.39 (0.44-4.42)	0.578
**Headache**										

No	1.00 (Reference)				1.00 (Reference)				1.00 (Reference)	
Yes	0.89 (0.63-1.25)	0.494			1.20 (0.60-2.11)	0.719			0.56 (0.22-1.41)	0.217
**Sleepiness during day**										
No	1.00 (Reference)		1.00 (Reference)		1.00 (Reference)				1.00 (Reference)	
Yes	2.28 (1.63-3.18)	<0.001	1.60 (1.06-2.42)	0.026	1.64 (0.92-2.93)	0.097			2.36 (0.98-5.70)	0.056
**Confusional state**										
No	1.00 (Reference)				1.00 (Reference)				1.00 (Reference)	
Yes	2.55 (1.51-4.33)	0.005			1.75 (0.77-3.99)	0.180			3.86 (0.67-22.4)	0.131
**Involuntary tremors**										
No	1.00 (Reference)				1.00 (Reference)				1.00 (Reference)	
Yes	2.97 (1.64-5.36)	<0.001			1.99 (0.80-4.84)	0.139			21.4 (2.57-178.0)	<0.001
**Tingling in the limbs/face**										
No	1.00 (Reference)				1.00 (Reference)				1.00 (Reference)	
Yes	1.15 (0.74-1.18)	0.5280			1.17 (0.57-2.43)	0.671			1.92 (0.73-5.62)	0.185
**Number of overall symptoms[2]**										
1	1.00 (Reference)				1.00 (Reference)				1.00 (Reference)	
2	2.82 (1.58-5.04)	<0.001			1.52 (0.53-4.34)	0.435			2.00 (0.39-10.2)	0.403
3	7.35 (4.06-13.3)	<0.001			3.30 (1.26-8.64)	0.015			7.00 (1.45-33.7)	0.015
≥4	15.0 (8.83-25.5)	<0.001			5.44 (1.89-15.6)	0.002			15.2 (3.68-63.1)	<0.001

OR =Odds ratio represent the probability of calling the GP over probability of not calling the GP

[1] In the table are reported only the statistically significant variables implemented in the multivariate model

[2] Not evaluated in multivariate analyses due to multicollinearity issues
